# Arrhythmogenic Biophysical Phenotype for SCN5A Mutation S1787N Depends upon Splice Variant Background and Intracellular Acidosis

**DOI:** 10.1371/journal.pone.0124921

**Published:** 2015-04-29

**Authors:** Rou-Mu Hu, Bi-Hua Tan, David J. Tester, Chunhua Song, Yang He, Sinisa Dovat, Blaise Z. Peterson, Michael J. Ackerman, Jonathan C. Makielski

**Affiliations:** 1 Department of Cardiology, China Meitan General Hospital, Beijing, China; 2 Department of Medicine, Division of Cardiovascular Medicine, University of Wisconsin, Madison, WI, United States of America; 3 Departments of Pediatrics, and Cellular & Molecular Physiology, Pennsylvania State University College of Medicine, Hershey, PA, United States of America; 4 Departments of Medicine, Pediatrics, and Molecular Pharmacology and Experimental Therapeutics, Mayo Clinic, Rochester, MN, United States of America; Rutgers-New Jersey Medical School, UNITED STATES

## Abstract

**Background:**

*SCN5A* is a susceptibility gene for type 3 long QT syndrome, Brugada syndrome, and sudden infant death syndrome. *I*_Na_ dysfunction from mutated SCN5A can depend upon the splice variant background in which it is expressed and also upon environmental factors such as acidosis. S1787N was reported previously as a LQT3-associated mutation and has also been observed in 1 of 295 healthy white controls. Here, we determined the in vitro biophysical phenotype of SCN5A-S1787N in an effort to further assess its possible pathogenicity.

**Methods and Results:**

We engineered S1787N in the two most common alternatively spliced SCN5A isoforms, the major isoform lacking a glutamine at position 1077 (Q1077del) and the minor isoform containing Q1077, and expressed these two engineered constructs in HEK293 cells for electrophysiological study. Macroscopic voltage-gated *I*_Na_ was measured 24 hours after transfection with standard whole-cell patch clamp techniques. We applied intracellular solutions with pH7.4 or pH6.7. S1787N in the Q1077 background had WT-like *I*_Na_ including peak *I*_Na_ density, activation and inactivation parameters, and late *I*_Na_ amplitude in both pH 7.4 and pH 6.7. However, with S1787N in the Q1077del background, the percentages of *I*_Na_ late/peak were increased by 2.1 fold in pH 7.4 and by 2.9 fold in pH 6.7 when compared to WT.

**Conclusion:**

The LQT3-like biophysical phenotype for S1787N depends on both the SCN5A splice variant and on the intracellular pH. These findings provide further evidence that the splice variant and environmental factors affect the molecular phenotype of cardiac SCN5A-encoded sodium channel (Na_v_1.5), has implications for the clinical phenotype, and may provide insight into acidosis-induced arrhythmia mechanisms.

## Introduction

The *SCN5A* gene encodes the voltage-gated cardiac Na^+^ channel (Na_v_1.5) also denoted SCN5A which is responsible for generating a large peak inward Na current (*I*_Na_) that underlies excitability and conduction in the working myocardium and specialized conduction tissue [1]. The SCN5A consists of a pore forming α-subunit composed of four homologous domains (I-IV), each containing six transmembrane segments (S1-S6). Alternative splicing of a glutamine (Q) at the beginning of exon 18 causes insertion of glutamine at position 1077 (Q1077), resulting in two splice variants, one containing 2,016 amino acids that is designated Q1077 and a 2,015-amino- acid protein that is designated Q1077del [2, 3]. Messenger RNA for these two splice variants was present in every human heart at a ratio of approximately 2:1 with the shorter 2,015-amino acid variant Q1077del predominant [2, 3]. The longer and less abundant Q1077 background was used in most studies of mutations in SCN5A [4].

**Fig 1:**
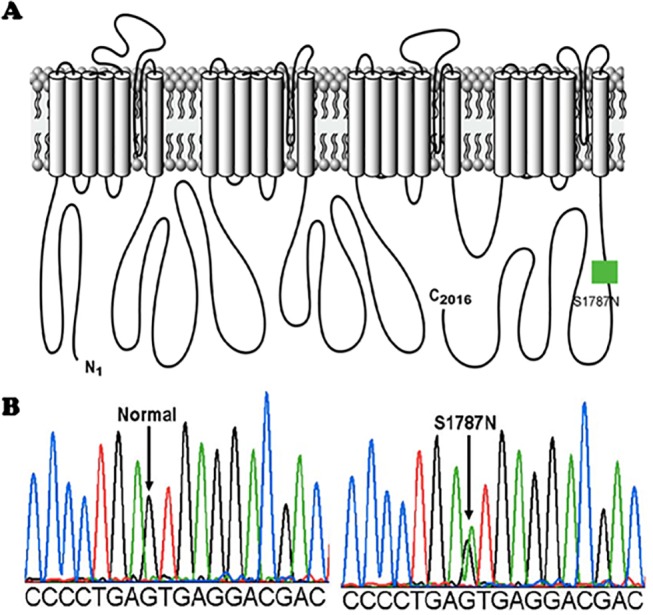
Topological diagram and sequence chromatogram of S1787N-SCN5A. (A) Topological diagram of SCN5A showing a missense mutation, S1787N on the linear topology of the C-terminus of cardiac Na channel. (B) Sequence chromatogram showing normal (left panel) and a missense mutation in codon 1787 of SCN5A resulting in a replacement of a serine (S) by an asparagine (N) (S1787N).

Mutations in SCN5A that pathologically increase late *I*_Na_ may influence repolarization and refractoriness. This “gain-of-function” effect on SCN5A causes type 3 long QT syndrome (LQT3) [5, 6]. *I*_Na_ dysfunction from mutated SCN5A can depend upon the splice variant background in which it is expressed [7] and also upon environmental factors such as acidosis [8]. S1787N was reported previously to be associated with LQT3 [9] and in 1 of 295 healthy white controls [10]. S1787N is located within the C-terminus of SCN5A (Fig 1) which has been shown to be important for sodium channel inactivation [11], but the dysfunction, if any, caused by this mutation has not been previously studied. Here, we determined the in vitro biophysical phenotype of S1787N in an effort to further assess its possible pathogenicity.

## Materials and Methods

### Site-directed mutagenesis and heterologous expression

The S1787N mutation was created by site-directed mutagenesis (mutagenesis kit from Stratagene) using a PCR technique. The appropriate nucleotide changes for S1787N were engineered into two common splice variants of SCN5A [one lacks glutamine at position 1077 (Q1077del; GenBank accession no.AY148488) and the other includes Q1077 (Q1077; GenBank accession no.AC1377587)] in the pcDNA3 vector (Invitrogen, Carlsbad, CA). The integrity of the constructs was verified by DNA sequencing to confirm the presence of the introduced mutation and the absence of Taq polymerase-induced substitutions that may occur during PCR. Wild-type (WT) or mutant channels in these two splice variants of SCN5A and another plasmid containing the construct for the green fluorescent protein (GFP) were transiently co-transfected into HEK 293 cells at a ratio of 5:1 with FuGENE6 reagent (Roche Diagnostics, Indianapolis, IN) according to manufacturer’s instructions [12, 13].

### Standard electrophysiological measurements

Macroscopic voltage-gated *I*_Na_ was measured 24 hours after transfection with the standard whole-cell patch clamp technique at a temperature of 22–24°C in HEK 293 cells expressed the GFP (‘green cells’). Cells were continuously perfused with bath (extracellular) solution containing 140 mM NaCl, 4 mM KCl, 1.8 mM CaCl_2_, 0.75 mM MgCl_2_ and 5 mM HEPES (pH 7.4 set with NaOH). The pipette (intracellular) solution contained 120 mM CsF, 20 mM CsCl_2,_ 5 mM EGTA and 5 mM HEPES and was adjusted to pH 7.4 with CsOH or pH 6.7 with CsOH. Microelectrodes were made of borosilicate glass with a puller (P-87, Sutter Instrument Co, Novato, CA, USA) and were heat-polished using a microforge (MF-83, Narishige, Tokyo, Japan). The resistances of microelectrodes ranged from 1.0 to 2.0 MΩ when filled with recording solution. Voltage clamp was generated by Axopatch 200B amplifier (Axon Instruments, Foster City, CA) and controlled using pClamp software 10.2. The series-resistance was compensated. Membrane current data were digitized at 100 kHz, low-pass filtered at 5 kHz, and then normalized to membrane capacitance. The standard voltage clamp protocols are presented with the data and have been previously described [2, 7, 14].

### Statistical analysis

All data points are shown as the mean value with the standard error of the mean (SEM). Determinations of statistical significance were performed using a Student’s t-test for comparisons of two means or using One-way ANOVA for comparisons of multiple means. A P value of < 0.05 was considered statistically significant. Curve fits were performed using pClamp 10.2 (Axon Instuments). Nonlinear curve fitting was performed with Origin 7.0 (Microcal Software, Northampton, MA, USA).

## Results

### Current expression and voltage-dependent gating of S1787N in Q1077del and Q1077 background

**Fig 2:**
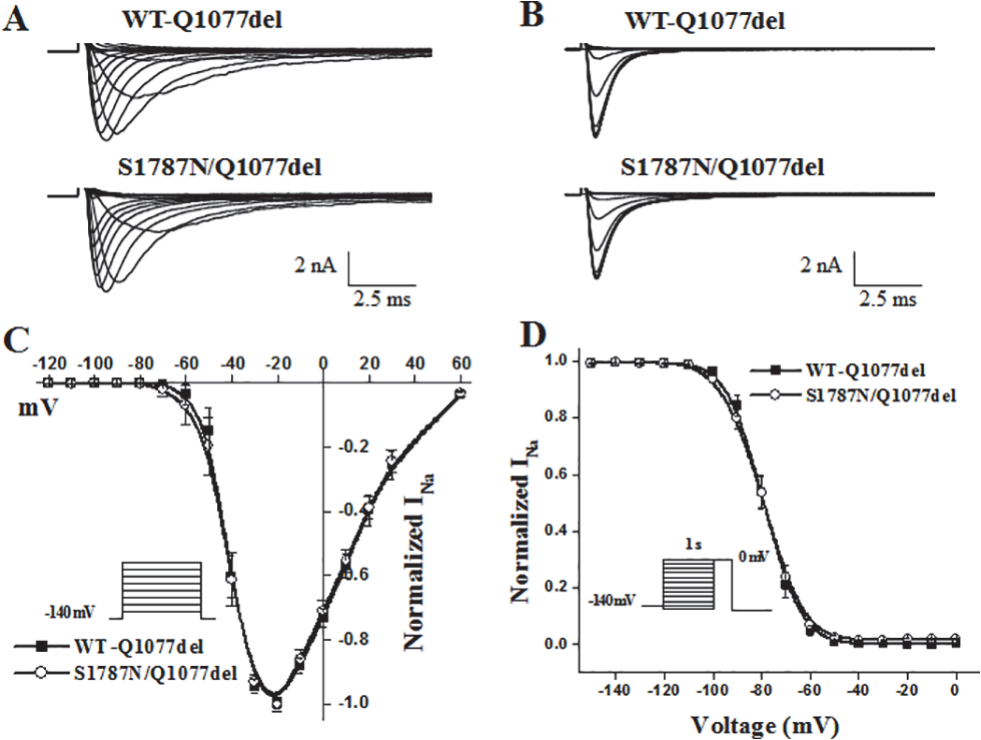
Electrophysiological properties of WT-Q1077del and S1787N/Q1077del. (A) Whole-cell current traces from representative experiments of WT-Q1077del and S1787N/Q1077del recorded for a peak current voltage relationship protocol (diagram inset in panel C) at membrane potentials between -120 to +60 mV in 10-mV increments from a holding potential of -140 mV. (B) Whole-cell current traces from representative experiments of WT-Q1077del and S1787N/Q1077del obtained in response to a steady state inactivation protocol (diagram inset in panel D) test depolarization to 0 mV for 24 ms from a holding potential of -140 mV, following 1s conditioning step to the various conditioning potentials. (C) Summary data for the peak current voltage relationship and (D) steady state inactivation with the line representing a fit to a Boltzmann equation. Parameters of the fits (midpoint V_1/2_ and slope K) and n numbers are reported in Table 1.

**Fig 3:**
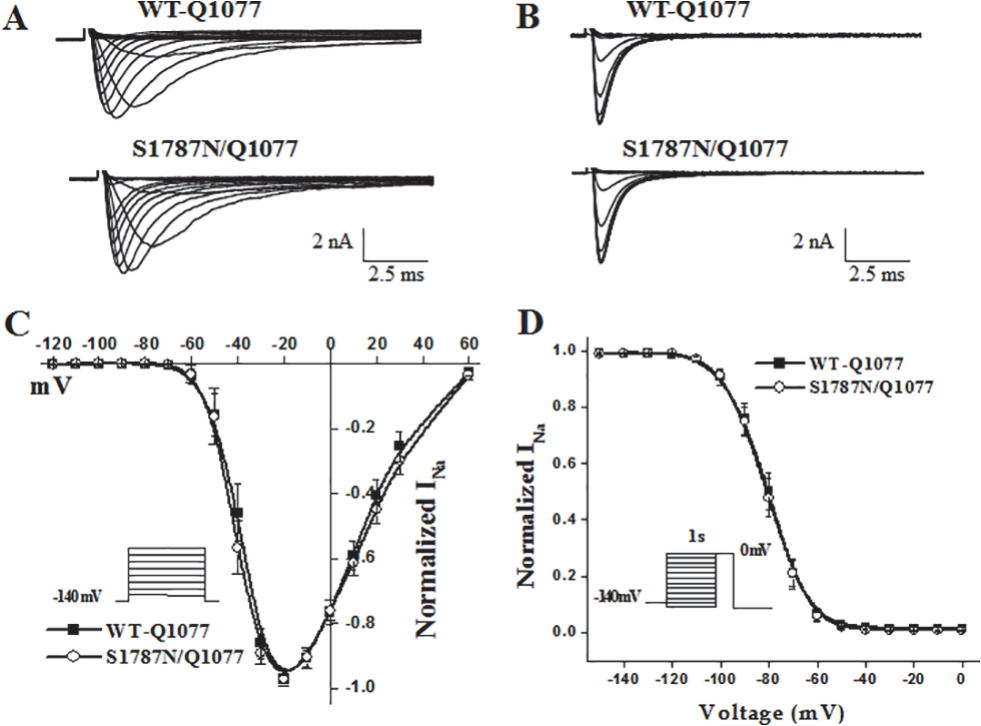
Electrophysiological properties of WT-Q1077 and S1787N/Q1077. (A, B) Whole-cell current traces and (C, D) summary data for peak current relationships and steady-state inactivation as described for Fig 2 but for experiments using WT-Q1077 and S1787N/Q1077 (see also Table 1).

WT and S1787N mutant channels in the two common splice variant backgrounds Q1077del and Q1077 were voltage clamped 24 hours after transient transfection with equal amounts of cDNA. Mean *I*_Na_ density for WT and mutant channels were compared for experiments performed on the same day in order to reduce variability. Examples of macroscopic *I*_Na_ traces for WT and mutant channels are shown in Figs 2 and 3 and summary data are given in Table 1. In the Q1077del background, the mean current density of S1787N mutant channel was 281 pA/pF, which was not significantly different from 295 pA/pF current density of WT (Table 1 and Fig 2A and 2B). In the Q1077 background, S1787N had a mean current density of 359 pA/pF, showing no significant difference compared with 301 pA/pF of WT (Table 1 and Fig 3A and 3B). In the Q1077del background, activation midpoint and inactivation midpoint of S1787N were no different than WT (Table 1 and Fig 2C and 2D). In the Q1077 background, S1787N also showed no difference in steady-state activation or inactivation parameters (Table 1 and Fig 3C and 3D).

**Table 1:** Voltage-dependent gating parameters of S1787N and WT in the Q1077del and Q1077 backgrounds.

Samples	Peak *I*_Na_ (pA/pF)	Activation		Inactivation V_1/2_ (mV)
		V_1/2_ (mV)	K	
WT-Q1077del	-295 ± 53 (19)	-41 ± 1.3	4 ± 0.3 (9)	-80 ± 1.6 (19)
S1787N/Q1077del	-281 ± 40 (18)	-42 ± 1.6	4 ± 0.2 (8)	-79 ± 2.1 (15)
WT-Q1077	-301 ± 34 (20)	-41 ± 2.4	4 ± 0.3 (13)	-80 ± 2.5 (14)
S1787N/Q1077	-359 ± 58 (19)	-42 ± 2.4	4 ± 0.2 (9)	-81 ± 2.0 (15)

The fitted values of voltage-dependent gating parameters represent the mean SEM for number of experiments in the parentheses. These parameters were obtained from fitting the individual experiments as in Figs 2 and 3 (C, D) to the appropriate model equations. For the Boltzmanm fits the parameters of V_1/2_ are the midpoint of activation and inactivation, and K is the slope.

### Late *I*_Na_ of S1787N in Q1077del and Q1077 background

**Fig 4:**
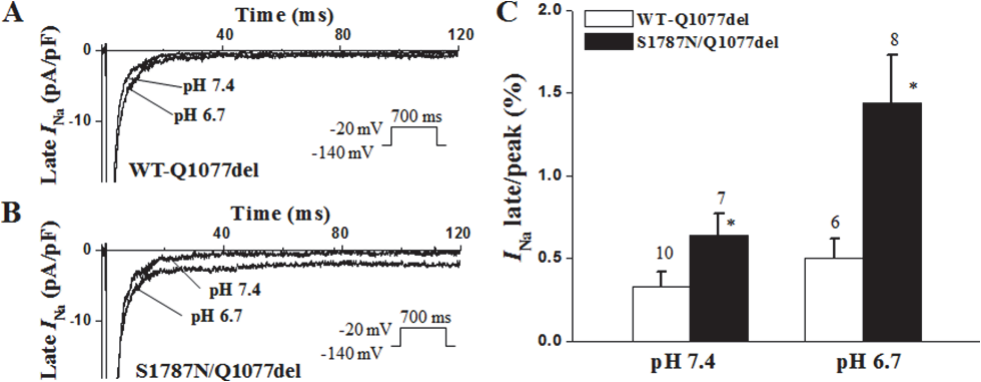
Late *I*_Na_ for S1787N/Q1077del and WT-Q1077del with pH 7.4 and pH 6.7. Representative current traces of WT-Q1077del (A) and S1787N/Q1077del (B) with pH 7.4 and pH 6.7 elicited by a test depolarization pulse from -120 mV to -20 mV for 700 ms (here only 120 ms shown). *I*_Na_ traces were normalized to cell capacitance and represented in pA/pF. (C) Summary data for late *I*_Na_ normalized to peak *I*_Na_. After leak-subtraction, the late *I*_Na_ was measured as the mean between 600 ms and 700 ms after the initiation of the depolarization. The number of experiments is indicated above the bar. *p< 0.05 indicated late *I*_Na_ of S1787N/Q1077del was increased significantly with pH 6.7 compared to pH7.4 and WT-Q1077del with pH7.4 and pH 6.7.

**Fig 5:**
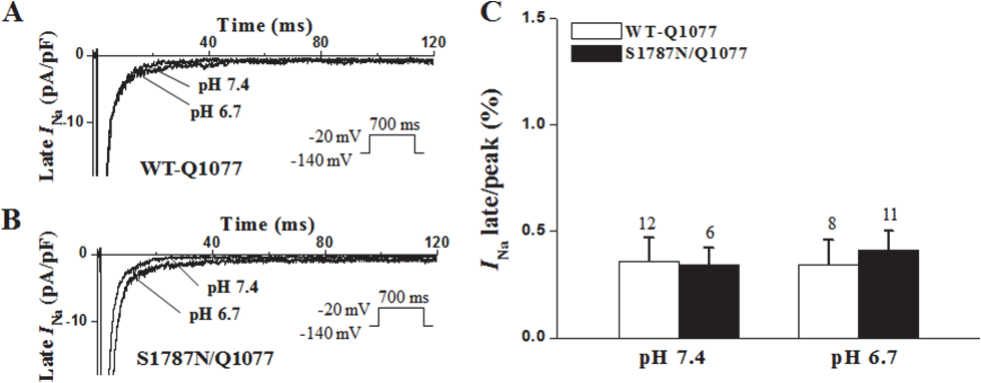
Late *I*_Na_ for S1787N/Q1077 and WT-Q1077 with pH 7.4 and pH 6.7. Representative late current traces of WT-Q1077 (A) and S1787N/Q1077 (B) and summarized percentage of late *I*_Na_ normalized to peak *I*_Na_ (C) under pH 7.4 and pH 6.7 as in Fig 4.

Late *I*_Na_ for both the S1787N mutant and WT channels in the two backgrounds was measured as the leak subtracted inward current remaining at the end of a 700-ms-long depolarization and expressed as a ratio of late/peak *I*_Na_ as previously reported [15, 16]. In the Q1077del background, late *I*_Na_ was increased by 2.1 fold for S1787N compared to WT channels (Fig 4, n = 7–9, p<0.05). However, when expressed in the Q1077 background, S1787N mutant channels exhibited similar levels of late *I*_Na_ as WT channels (Fig 5).

### Late *I*_Na_ for S1787N with intracellular acidosis in Q1077del and Q1077 background

We lowered the intracellular pH from 7.4 to 6.7 and measured late *I*_Na_ for S1787N mutant channels and WT channels in both Q1077del and Q1077 background. Interestingly, intracellular acidification (pH 6.7) evoked even greater significantly increased late *I*_Na_ for S1787N mutant channels when expressed in the Q1077del background (Fig 4). In the Q1077del background, the percentage of *I*_Na_ late/peak of S1787N was increased by 2.1-fold compared to that of WT in pH 7.4 (n = 7–9, p<0.05) and was increased by 2.9-fold compared to that of WT in pH 6.7 (n = 6–8, p<0.03), indicating intracellular acidosis exacerbated late *I*_Na_ for mutant channels expressing in the Q1077del background. In contrast, low intracellular pH caused only subtle change to late *I*_Na_ for S1787N mutant channels expressed in the Q1077 background compared with that of WT channels with pH 6.7 (Fig 5).

## Discussion

### The role of C-terminus in Na^+^ channel inactivation

Fast inactivation is due to rapid block of the inner mouth of the channel pore by the cytoplasmic linker between domains III and IV of SCN5A that occurs within milliseconds of membrane depolarization [17, 18]. Mutagenesis studies of this region revealed a hydrophobic isoleucine-phenylalanine-methionine (IFM) motif, which serves as a tethered pore blocker that binds to a receptor in the intracellular mouth of the pore, resulting in channel inactivation subsequent to channel opening [19, 20]. KPQ1505-1507 (ΔKPQ), a three-amino acid deletion mutation of the III-IV loop was identified as the LQT3 mutation causing persistent inward *I*_Na_ [1, 21]. The non-inactivating component of *I*_Na_ named late *I*_Na_ acts to prolong the plateau of the action potential (AP) allowing for the development of arrhythmogenic triggered activity referred as early afterdepolarizations (EADs) and predispose patients to torsade de pointes leading to syncope and sudden death [22].

The SCN5A C-terminus also has been shown to play a role in inactivation through chimeric studies and characterization of several disease-linked mutations are found in the C-terminus [23, 24]. Notably, gene disorders associated with LQT3 located in this region disrupt inactivation in a manner similar to mutations that affect the DIII/DIV linker inactivation gate [25, 26]. Mutations in this region also have been shown to underlie Brugada Syndrome (BrS) and sudden infant death syndrome (SIDS). The E1784K mutation has been found in LQTS patients [27–29]. The affected glutamic acid residue (E1784) is located within a highly conserved acidic domain immediately following the D4/S6 segment [28]. Functional characterization of E1784K revealed a persistent inward current attributed to late *I*_Na_ and a faster recovery from inactivation indicating a destabilization of the inactivation state [27]. Another mutation located in the C-terminus was identified as 1795insD [25, 30]. 1795insD disrupted fast inactivation causing late *I*_Na_ and appeared as the LQT3 phenotype at slow heart rates [25]. Makita et al. discovered a novel mutation located at the C-terminal named L1825P exhibiting biophysical properties such as prominent late *I*_Na_ and decreased current density, which are strikingly similar to 1795insD [31]. Another nearby mutation R1826H associated with SIDS conferred a “gain-of-function” phenotype characterized by late *I*_Na_ similar to other reported LQT3-associated mutations [32]. It has been suggested that calmodulin (CaM) may modulate the interaction between the C-terminus and III-IV linker [33]. Disruption of the complex by mutation thus removes this physiologically key structural component of the channel and allows for “leakage” of Na^+^ into cells under conditions that normally exclusively prevent Na^+^ entry [34]. Furthermore, biochemical and biophysical evidence support the presence of a predicted EF-hand motif in the proximal C-terminus of the SCN5A and demonstrate the importance of its stabilization by an interhelical hydrophobic interface, of which perturbation results in the destabilization of sodium channel inactivation [11].

In our study, the S1787N variant, located within the SCN5A C-terminus, is derived from a G-to-A substitution at nucleotide 5360 resulting in a replacement of an uncharged serine (S) by negative-charged asparagine (N) at amino acid position 1787. This transition may affect conformational structure of the C- terminus and disturb the III-IV linker-C-T interaction, thus destabilizing sodium channel inactivation and promoting persistent inward late *I*_Na_.

### Effect of alternative splice variants of SCN5A on Na^+^ channel

Functional studies of WT Na^+^ channels showed no significant difference between Q1077del and Q1077 background in functional properties [2]. However, when mutations are introduced into these two splice backgrounds, electrophysiological discrepancies of Na^+^ channels were found. Wang et al. discovered one in-frame deletion allele (delAL586-587) and two missense variants (R680H, V1951L) that exhibited increased persistent sodium current only when expressed in the context of the common splice variant Q1077del [35]. Tan et al. found a BrS-associated mutation G1406R that caused a partial trafficking defect with slowed and incomplete expression and the severity of the defect depended on the background splice variant in which it is expressed, being worse in the Q1077 variant [3]. The missense variant R1193Q was found to cause “loss-of-function” in the Q1077 background and “gain-of-function” in the Q1077del background, leading to BrS and LQT3 phenotypes respectively [36, 37]. Our study shows that S1787N significantly increased late *I*_Na_ only in the splice variant Q1077del. This result again demonstrate that the splice variant background can be important in determining the functional properties of a mutation in heterologous expression systems, and this could be one explanation for the clinical observations of the same single SCN5A missense mutation leading to different phenotypes, depending on the different genetic background.

### Effect of intracellular pH on Na^+^ channel

Intracellular acidosis evoked a significantly greater level of increased late *I*_Na_ in cells expressing S1787N with Q1077del. Previous studies by Plant et al. [8] and Wang et al. [35] have shown similar results. The S1103Y SCN5A polymorphism found in African-Americans has been linked to lethal arrhythmias in families with ventricular tachycardia [38]. Intracellular acidosis (pH 6.7) increased late *I*_Na_ for S1103Y and R680H in a case of autopsy negative sudden unexplained death and suggested this as an etiologic factor [39]. We hypothesize that increased risk for arrhythmia in patients bearing S1787N/Q1077del may result from a similar mechanism. It is reported that intracellular pH of cardiac myocytes is lower than normal arterial pH which is approximately 7.4 [40, 41]. Internal pH may decrease to 6.6–6.8 with respiratory acidosis and/or hypoxia and basal microheterogeneity in intracellular pH would be pronounced under stress due to slow dissipation of acid through myocardium [42, 43]. Therefore, the environmental factor of acidosis could be a trigger for arrhythmia.

### Pathogenecity of S1787N

The biophysical phenotype of increased late *I*_Na_ we describe provides a plausible arrhythmogenic mechanism for the type 3 long QT syndrome. However, this allele also occurs in apparently healthy population which brings pathogenicity into question. Our data indicate that the abnormal biophysical phenotype of the S1787N depends on the different splice variant backgrounds and the environmental trigger of acidosis, suggesting that S1787N requires the double hit of genetics and environment to manifest a clinical phenotype. It must be pointed out that our functional study has limitations. The experiments were performed by expressing the channels in heterologous cell culture (HEK-293 cells). Heterologous systems do not faithfully recapitulate the actual cellular phenotypes because they do not have the full panoply of subunits and regulator machinery found in the native heart cells. Nonetheless, this is the classic and standard technique used for all previous studies of SCN5A function of mutant ion channels, including putative LQT3 and BrS mutations. Despite this limitation we have described current dysfunction that is plausibly pathogenic. It is important to note, however, that undiscovered genetic, developmental, or environmental factors could underlie the pathogenicity, either in concert with the S1787N variant, or independently.

## Conclusion

In conclusion, this study characterized the abnormal biophysical phenotype of the SCN5A variant, S1787N, depended on the splice variant background Q1077del and the environmental factor such as intracellular acidosis. These findings emphasize the importance of characterizing the biophysical function of SCN5A arrhythmia mutations using the two common background channel sequences. Furthermore, evidence that the splice variant and environmental factors affect the molecular phenotype of cardiac SCN5A-encoded sodium channel (Na_v_1.5) has implications for the clinical phenotype and may provide new insight into acidosis-induced arrhythmia mechanisms.
